# *Ganoderma neo-japonicum* Imazeki revisited: Domestication study and antioxidant properties of its basidiocarps and mycelia

**DOI:** 10.1038/srep12515

**Published:** 2015-07-27

**Authors:** Wee-Cheat Tan, Umah Rani Kuppusamy, Chia-Wei Phan, Yee-Shin Tan, Jegadeesh Raman, Azliza Mad Anuar, Vikineswary Sabaratnam

**Affiliations:** 1 Mushroom Research Centre, University of Malaya, 50603 Kuala Lumpur, Malaysia; 2 Institute of Biological Science, Faculty of Science, University of Malaya, 50603 Kuala Lumpur, Malaysia; 3 Department of Biomedical Science, Faculty of Medicine, University of Malaya, 50603 Kuala Lumpur, Malaysia; 4 Centre of Excellence for Learning and Teaching, UCSI University, Jalan Menara Gading, Taman Connaught, 56000 Kuala Lumpur, Malaysia

## Abstract

Mushroom cultivation benefits humankind as it deliberately encourages wild mushrooms to be commercially propagated while recycling agricultural wastes. *Ganoderma neo-japonicum* is a rare polypore mushroom found growing on decaying *Schizostachyum brachycladium* (a tropical bamboo) clumps in Malaysia. The Malaysian indigenous tribes including the Temuans and Temiars use the basidiocarps of *G. neo-japonicum* to treat various ailments including diabetes. In this study, the domestication of *G. neo-japonicum* in artificial logs of different agricultural residues was investigated. Sawdust promoted the mycelia spawn colonisation in the shortest period of 38 ± 0.5 days. However, only sawdust and bamboo dust supported the primodia formation. Complex medium supported mycelium growth in submerged cultures and 27.11 ± 0.43 g/L of mycelia was obtained after 2 weeks of cultivation at 28 °C and 200 rpm. Antioxidant potential in mushroom may be influenced by different cultivation and extraction methods. The different extracts from the wild and cultivated basidiocarps as well as mycelia were then tested for their antioxidant properties. Aqueous and ethanol extracts of mycelia and basidiocarps tested had varying levels of antioxidant activities. To conclude, domestication of wild *G. neo-japonicum* using agroresidues may ensure a continuous supply of *G. neo-japonicum* for its medicinal use while ensuring the conservation of this rare species.

*Ganoderma* spp., in particular *G. lucidum* (Curtis: Fr.) P. Karst have a long history in traditional Chinese medicine. The mushroom, also known as Lingzhi has been widely prescribed for prevention and treatment of many chronic diseases including hypertension, bronchitis, arthritis, neurasthenia and neoplasia^1^. The curative effects of *Ganoderma* spp. are attributed to their superior antioxidant properties^2^. Natural products and food-derived antioxidants have received great attention because of their chemoprevention properties against oxidative damages such as inflammation^3^. Inflammation of normal cells caused by over-production of reactive oxygen species (ROS) has been identified as the main factor causing nearly all life-threatening diseases such as cancer, cardiovascular and neurological disorders^4^. Therefore, antioxidants that can remove excess ROS are important to protect the human body against oxidative stress. Moreover, natural antioxidants are considered safer when compared to synthetic antioxidants. In this regards, *Ganoderma* mushrooms are good candidates as antioxidative agents because they are natural and rich in polysaccharides, bioactive components such as terpenes, ganoderic acids, and phenolic compounds^5^,^6^. *Ganoderma lucidum* has been well investigated and several of the ethnomycological claims of its curative properties have been scientifically validated^7^. There are, however, several neglected *Ganoderma* spp. which may add to the medicinal properties knowledge pool leading to new drug or nutraceutical discoveries^8^,^9^.

One such species available from the forest is *Ganoderma neo-japonicum* Imazeki which is a rare saprotrophic and annual *Ganoderma* species colonising decaying bamboo clumps. This mushroom is distributed in Asian countries including China, Korea, Japan, Taiwan and Malaysia. In Malaysia, *G. neo-japonicum* which grows in the forests and only on decaying clumps of tropical bamboo (*Schizostachyum brachycladium*) has been harvested by the indigenous people (*Orang Asli*) for its medicinal properties. *Orang Asli* is the term specifically used in referring to the indigenous people living in Peninsular Malaysia. There are three main groups namely the Negrito, Proto-Malays and Senoi. Each of these groups have six tribes and there are 150,000 *Orang Asli* in Malaysia^10^,^11^. The medicinal uses of *G. neo*-*japonicum* based on an on-going study to record the tacit knowledge of the use of mushrooms as food and medicine are summarised in Table 1 8,9,12,13,14,15,16,17,18,19,20. The basidiocarp was boiled in water and the decoction was consumed to treat fever, asthma, diabetes, joints and body aches as well as a tonic to improve body strength. The stipe was cut into bead-like pieces, strung and worn round the neck of children to treat epilepsy.

However, the scientific validation of the medicinal value of this mushroom has not been extensively investigated. To date, *G. neo-japonicum* has been reported as a potent radical scavenger and showed hepatoprotective activity *in vivo*^17^. Hirotani *et al.*^19^ isolated and characterised two drimane sesquiterpenes, namely cryptoporic acids H and I from the basidiocarps of *G. neo-japonicum*. Two lanostanoids (ganoderal A and ganodermadiol), steroid (2*β*,3α,9α-trihydroxyergosta-7,22-diene) and four ergosteroids were also reported from this mushroom^16^. In our studies, we reported that wheat grains fermented by mycelia of *G. neo-japonicum* had enhanced antioxidant activities as well as enhanced adipogenesis; and that PPARγ expression in 3T3-L1 cells was modulated^9^,^20^. Further, the aqueous extracts of *G. neo-japonicum* had a significant (p < 0.001) effect on neurite outgrowth stimulatory activities when compared to nerve growth factor, the positive control^8^. Table 2 summarises the studies on the medicinal properties of *G. neo-japonicum* for the last two decades.

Mushrooms can be artificially cultivated for mycelia biomass production^21^. There are, however, minimal reports on artificial cultivation for basidiocarp production^21^,^22^ and solid substrate fermentation (SSF) of *G. neo-japonicum*^20^. Besides the basidiocarp, mycelia of mushrooms can also be obtained *via* SSF. This process has been reported to enhance the phenolic content and antioxidant potential in fermented foods^23^. In SSF process, non specific hydrolytic enzymes were secreted to degrade lignocellulose, thus releasing various phenolic compounds. Chemical composition of *Cordyceps sinensis* and the bioactivity of stale rice were improved by SSF as reported by Zhang *et al.*^24^. The nutritional quality and antioxidant activity of different agro-residues such as cocoa pod husk, cassava peels and palm kernel cake were also enhanced by SSF^16^.

*Ganoderma neo-japonicum* is not abundantly available in the forest due to the annual growth cycle of this species in nature. Mycelium production *via* SSF may provide an alternative way to overcome the limited supply of *G. neo-japonicum* and for conservation of this invaluable, rare polypore both in and *ex situ*. Therefore, the aims of the study were to revisit *G. neo-japonicum* by optimising the cultivation conditions of *G. neo-japonicum* by different formulation of selected agricultural wastes as well as cultivation of the mycelium via fermentation. The antioxidant activities of the various extracts prepared from basidiocarp (wild and cultivated) and mycelium from submerged and solid substrate fermentation were also investigated.

## Results

### Selection of substrate for basidiocarp production based on mycelia growth

**Figure 1:**
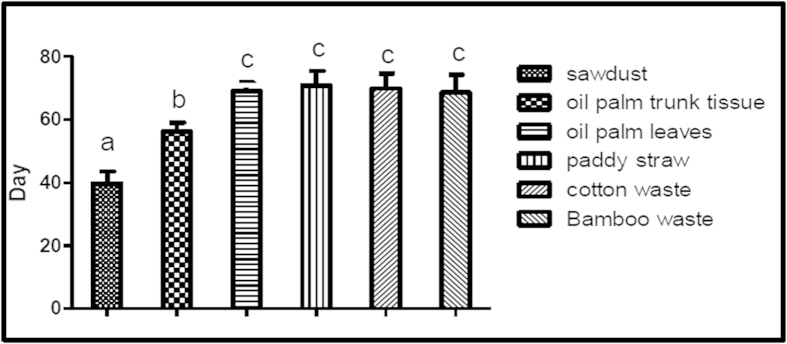
The effects of different agro-residues on the mycelia growth of *Ganoderma neo-japonicum*. The growth was measured in days taken to colonise 500 g of substrate using 5% (w/w) of 7-day inoculum at 28 ± 2 °C. Results with the similar alphabets are not significant different at p < 0.01.

All lignocellulosic substrates tested supported mycelia growth (Fig. 1). The support of mycelia growth and colonisation of the different substrates was in descending order: sawdust > oil palm trunk tissue > oil palm leaves ≥ bamboo waste ≥ cotton waste ≥ paddy straw. The growth rate of *G. neo-japonicum* was the highest (p < 0.05) on sawdust whereby 39.8 ± 1.2 days was taken to colonise a 500 g mushroom bag (Fig. 1).

### Mushroom basidiocarp production

**Figure 2:**
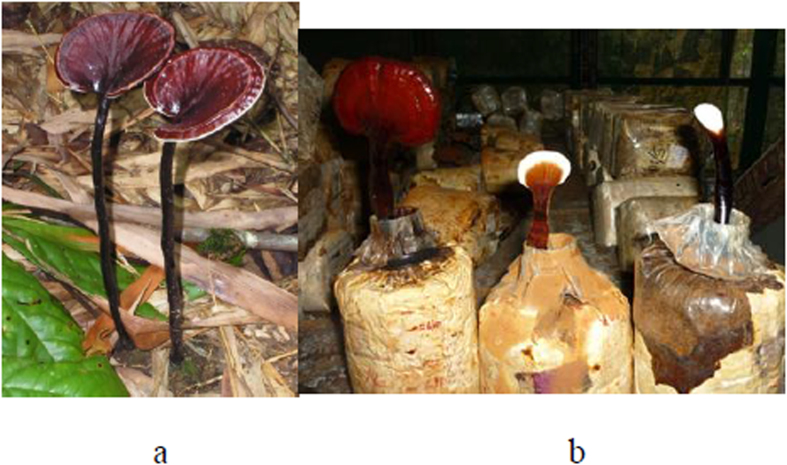
(**a**) Wild mature basidiocarp and (**b**) Different growth stages of domesticated *Ganoderma neo-japonicum* growing on rubber tree sawdust.

**Figure 3:**
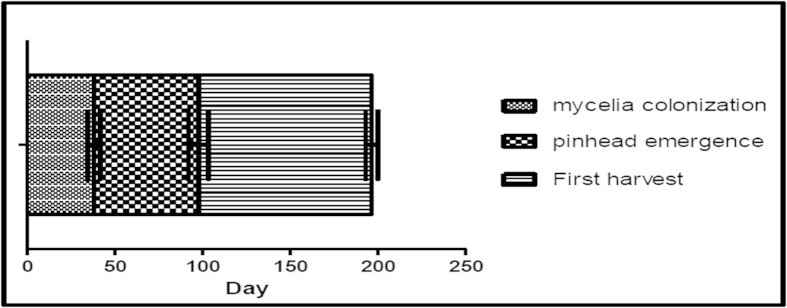
Life cycle of *Ganoderma neo-japonicum* in artificial cultivation consisting of 500 g of substrates. 5% (w/w) of 7-day inoculum was used and incubated at 28 ± 2 °C and 25 ± 3 °C for spawn run and primodia development, respectively.

Sawdust was selected as the substrate for preliminary domestication of *G. neo-japonicum* and basidiocarp production since it supported the highest growth rate of mycelia. The duration of a complete colonisation was about 38.0 ± 0.5 days. The duration recorded for pinhead emergence and maturation of basidiocarp in the first harvest was 59.7 ± 1.1 and 98.7 ± 0.7 days, respectively (Figs 2 and 3).

### The effects of media on mycelia production in SmF

**Figure 4:**
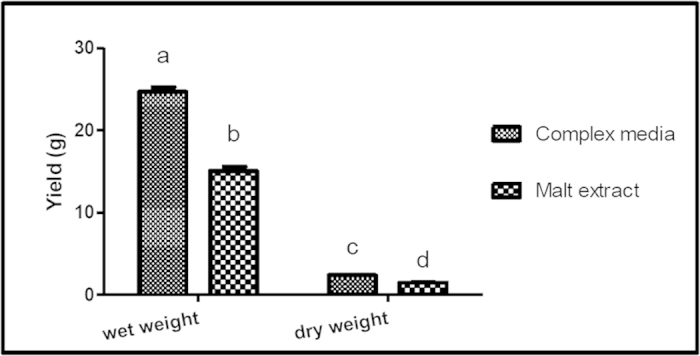
Biomass production of *Ganoderma neo-japonicum* in malt extract and complex media. Results with the similar alphabet are not significant at p < 0.05.

The mycelia production of *G. neo-japonicum* in the complex media (24.68 ± 0.58 g/L) was 1.6-fold higher than the mycelia produced in malt extract broth (15.07 ± 0.51 g/L) (Fig. 4). The mycelia production was comparable to the 21.53 ± 0.38 g/L of mycelia produced by *G. lucidum*^25^.

However, the dry cell weight (DCW) harvested after freeze drying was only about 10% of the fresh mycelia. Glucose and peptone present in the complex media may have promoted mycelia production.

### The effects of shaking speeds on mycelia production in SmF

**Figure 5:**
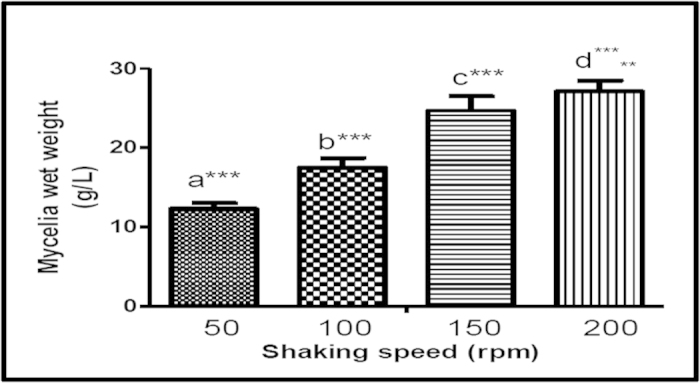
The effects of shaking speed on biomass production of *Ganoderma neo-japonicum*’s mycelia in complex media. Results with the same alphabet are not significant. ***at p < 0.01, **at p < 0.05.

The effects of shaking speed (50, 100, 150 and 200 rpm) in the mycelia production in submerged culture were further investigated. The shaking speed of 200 rpm gave the highest yield of mycelia (27.11 ± 0.43 g/L), followed by the shaking speed of 150 rpm (24.68 ± 0.58 g/L), and 100 rpm (17.60 ± 0.38 g/L) (Fig. 5). The lowest yield was at 50 rpm (12.32 ± 0.22 g/L).

### Quantification of total phenolic content (TPC) and antioxidant activity of different extracts of *G. neo-japonicum*

Total phenolic content was found to be the highest in ethanolic extract of wild basidiocarp (101.01 ± 0.59 μgGAE/mg), followed by hot aqueous extract of cultivated basidiocarp (23.76 ± 0.76 μgGAE/mg) (Table 3). Ethanolic extract of wild basidiocarp showed the highest DPPH quenching effects with IC_50_ value 29.95 μg/ml (Table 3). The radical scavenging ability of the different extracts decreased in the following order: ethanolic extract of wild basidiocarp > hot water extract of wild basidiocarp > ethanolic extract of SSF > hot water extract of mycelia > ethanolic extract of mycelia > filtrate > hot water extract of cultivated basidiocarp > ethanolic extract of cultivated basidiocarp. Similarly, the ethanolic extract of wild basidiocarp also showed the highest ferric-reducing power (0.76 ± 0.03 mol Fe^2+^/g), followed by hot water extract of wild basidiocarp (0.36 ± 0.01 mol Fe^2+^/g). In ABTS^•+^ decolourisation assay, the results were comparable to those obtained in DPPH and ferric-reducing reactions. All the scavenging activities were dose-dependent, whereby the highest ABTS^•+^ scavenging was observed for ethanolic extract of wild basidiocarp (720.85 μg/ml), followed by hot water extract of cultivated basidiocarp (1343.01 μg/ml) and hot water extract of wild basidiocarp (1801.74 μg/ml).

## Discussion

As shown in Table 4, the higher lignin content in sawdust (14–34%) and oil palm trunk tissue (22.6%) may have enhanced the growth rate of mycelia^26^,^27^,^28^,^29^,^30^,^31^. *Ganoderma neo-japonicum*, a white rot fungus, produces different types of extracellular enzymes to aid in colonising a specific substrate^32^. *G. neo-japonicum* showed strong β-glucosidase and avicelase activities and moderate ligninase activity^12^,^13^. Further, *G. neo-japonicum* has been reported to produce extracellular oxidative enzymes including laccase and lignin peroxidases which may aid in the degradation of lignocellulose-based substrates^17^.

Although the growth cycle of this species is much longer compared to the other well domesticated *G. lucidum* and *G. tsugae*, it was still shorter as compared to the reported annual life cycle in the wild^33^. For the production of basidiocarp, the sawdust with an adjusted moisture content of 70% was supplemented with 10% (w/w) rice bran and 1% (w/w) calcium carbonate. Moisture content in substrate is very important for enzymatic hydrolysis processes during mycelia growth, while rice bran provides the organic nitrogen, essential vitamins B and trace elements to boost mycelia growth. Calcium carbonate functions by maintaining a near neutral pH in mushroom bags. It acts as a buffering system to prevent acidic environment due to accumulation of carbon dioxide throughout the incubation period. Shorter lag phase in growth cycle and a near neutral pH will minimise the contamination of mushrooms bags by other fungi, for example *Aspergillus* sp., *Trichoderma* sp. and *Rhizopus* sp.^34^.

Most basidiomycetes prefer complex organic nitrogen sources as selected essential amino acids may not be synthesised from inorganic nitrogen sources in the submerged culture^35^. Mycelia production increased with the shaking speed and this could be due to better aeration which is essential for cell growth. Improvement of efficiency and gradient of gas exchange, as well as reduction of carbon dioxide during agitation may also play important roles in submerged culture. Furthermore, higher shaking speed could inhibit pellet formation of mycelia and hence encourage a homogenous mycelium growth as suggested by Wagner *et al.*^36^.

Mushrooms are noted for their abundance of polar phenolic compounds especially the phenolic acids, for example gallic acid, tannic acid, protocatechuic acid, and gentisic acids^37^. In general, the wild growing mushrooms have higher concentration of phenolics as suggested by Grangeia *et al.*^38^. The hot aqueous extract of *G. lucidum* cultivated in Malaysia was found to contain 63.51 ± 3.11 mg GAE/g extract of TPC^39^. The amount of TPC was higher when compared to the hot aqueous extract of *G. neo-japonicum* (23.76 ± 0.76 μg GAE/mg). As reported by Park and Lee^14^, the TPC content of *G. neo-japonicum* was 5.78 ± 0.68 mg GAE/g dry weight. The TPC was significantly increased by 13.2-fold upon supplementation of an amino acid, tryptophan. As an aromatic amino acid, tryptophan might play a role in the shikimate pathway and phenylpropanoid pathway, hence regulated the TPC in mushrooms. Besides, we observed that the polarity of extraction solvent had a direct influence on the phenolic content of the resulting extracts. In this study, the ethanol extract revealed a higher TPC. On the other hand, Wong *et al.*^40^ showed that the hot aqueous extracts of *Hericium erinaceus* had a higher TPC than that of methanol extracts.

Our results showed that the extracts of wild basidiocarps showed higher free radicals scavenging effects as compared to the cultivated ones. We also found that solvent extraction by ethanol was the best extraction method to maximise the antioxidative activities of this mushroom. *Ganoderma* sp. has been shown to be effective in free radical scavenging and chelating. The hot aqueous extract of a locally cultivated *G. lucidum* exhibited significant DPPH scavenging activity with IC_50_ of 5.280 ± 0.263 mg/ml^39^. Our result indicated that the hot aqueous extract of *G. neo-japonicum* had stronger ability to scavenge free radical, hence a better antioxidant; since its IC_50_ value was lower (1343.01 ± 7.33 μg/ml). Park and Lee^15^ reported that the DPPH and ABTS scavenging effects of *G. neo-japonicum* mycelia extracts were 11.80 ± 0.48 mg/mL and 2.12 ± 0.23 mg/mL, respectively. The discrepancy between their results and ours was maybe due to different cultivation methods. Tseng *et al.*^2^ reported that polysaccharides extracted from the basidiocarp of *G. tsugae* exhibited a stronger antioxidant properties compared to those extracted from mycelia and filtrate. According to Bhanja *et al.*^23^, the production of β-glucosidase enzyme during fermentation process was responsible for polyphenol accumulation and radical scavenging properties. There was a significant difference in antioxidant activities across all assays of the extracts of wild basidiocarp when compared to the cultivated basidiocarp extract as well as extracts of mycelium and fermented grains. This observation has not been reported by other studies. The difference in biological activities among ethanolic and hot water extracts of mushrooms were attributed to the differences in their chemical composition, especially polysaccharides, and phenolic content^41^.

On-going studies show that *G. neo-japonicum* exhibited genoprotective and anti-inflammatory properties (unpublished data). This mushroom which is used in traditional medicine by the indigenous tribes of Malaysia may be developed as a nutraceutical and therapeutic agent. However, caution is to be taken to ensure that the domestication process retains the bioactivities of this mushroom. Besides, we also observed that the antioxidant activities of mycelia and fermented grains were not comparable to the activities detected in wild basidiocarp. Therefore, environmental and nutritional factors in the cultivation process need to be kept as close to the nature as possible. Since *G. neo-japonicum* is a traditionally used mushroom with promising potentials, further investigations on the domestication process was warranted.

*Ganoderma neo-japonicum* can be cultivated for basidiocarps as well as cultured in SSF with wheat grains or SmF with complex media tom obtain the mycelia. Among the different agro-residues tested, sawdust was the best substrate for cultivation of this mushroom. Complex medium and shaking speed of 200 rpm were found to support the maximum mycelia production. The ethanolic extract of wild basidiocarps showed the highest phenolic content and antioxidant activities when compared to the cultivated basidiocarps and mycelia in SSF and SmF. The loss of the antioxidant properties of *G. neo-japonicum* during the domestication processes was noted and the domestication process has to be optimised to retain the nutraceutical potential in the cultivated basidiocarps. Further, the cultivation of basidiocarps, not only will ensure a constant supply of the mushrooms for the tribes but also reduce over harvesting of the wild gene pool in the forests. As this mushroom also contributes to the economic activity of the tribes, cultivation on artificial logs may contribute to their income as well.

## Methods

### Mushrooms sampling and collection

Basidiocarps of *G. neo-japonicum* were collected by Mr Tan Wee Cheat during an expedition at Lata Kijang, Kenaboi Forest Reserve, Negeri Sembilan (2–5 May, 2010). The indigenous people (Temuan and Jahai tribes) live in these forests. The basidiocarps were air dried at room temperature prior to processing and a voucher specimen (KLU-M 1231) was deposited in the Herbarium Kuala Lumpur, University of Malaya. The identity of the mushroom was authenticated by Professor Yi-Jian Yao, Director of Fungarium (HMAS), Institute of Microbiology, Chinese Academy of Sciences, Beijing 100101, People’s Republic of China.

### Tissue culture of wild basidiocarp

A mycelium culture of the basidiocarp of wild *G. neo-japonicum* (KLU-M 1231) was established by tissue culture technique^34^. The mycelia were maintained on malt extract agar (MEA) at 10 °C.

### Mycelia growth and primordia formation by *G. neo-japonicum*

Bamboo waste, oil palm trunk tissue, oil palm leaves, paddy straw, cotton waste and sawdust were obtained from handicraft centres, oil palm plantation, paddy fields, cotton processing factory and sawmills, respectively. All the biomaterials were chopped into small size (0.1–0.5 cm), mixed with rice bran and calcium carbonate in 89:10:1 ratio and then mixed with water to obtain 70% moisture. Plastic bags were filled with 500 g of well mixed substrates, autoclaved at 121 °C and 15 psi for one hour. The mushroom bags were then left to cool overnight and then inoculated with 5% (w/w) of two-week-old fermented wheat grains prepared as mentioned in SSF below. The bags were incubated at 27 ± 2 °C in the dark^34^. Spawn (mycelia) run was recorded every three days. When the mycelia had been fully colonised the substrates, the bags were transferred to the mushroom house and high humidity (70–90%) and temperature (25 °C) were maintained to induce primordia formation. Yield was calculated as biological efficiency (%).
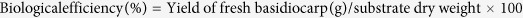


### Production of *mycelia* by submerged fermentation (SmF)

Five mycelia dics of *G. neo-japonicum* from 10-day-old cultures growing on malt extract agar (MEA, Difco) was/were inoculated into several 500 mL Erlenmeyer flask each containing 200 mL sterile complex seed medium (g/L): malt extract 8; yeast extract 8; peptone 8; glucose 15; NH_4_Cl 1; KH_2_PO_4_ and MgSO_4_.7H_2_O 1^25^. The inoculated flasks were incubated for seven days at 150 rpm and 28 ± 2 °C. The mycelium biomass from the seed culture (10 mL) was then inoculated into 2 L complex media in 5 L Schott Duran bottle. The mycelia were harvested after two weeks of incubation.

### Media type and shaking speeds on mycelia production

*Ganoderma neo-japonicum* mycelia production in SmF in two different media (malt extract and complex media) and shaking speeds (50, 100, 150, 200 rpm) were compared. Harvested mycelia were freeze dried (lyophilised) and kept in 4 °C for further assay.

### Production of mycelia by solid substrate fermentation (SSF)

Wheat grains were soaked overnight in distilled water and excess water was drained. About 250 g of soaked grains were dispensed into each of several 500 mL conical flasks and autoclaved at 121 °C, 15 psi for 15 minutes^42^. The flasks were then cooled to room temperature and inoculated with 3 discs of 10 days old mycelia of *G. neo-japonicum* grown on MEA plates. The fermented grains were then harvested after two weeks of incubation at room temperature and used to inoculate mushroom bags or freeze dried and kept for antioxidant analysis.

### Preparation of aqueous and ethanol extracts

Freeze dried mycelia, fermented wheat grains, and air dried basidiocarps (both wild and cultivated) were blended, soaked in 95% ethanol at 1: 100 (w/v) on 150 rpm shaker and incubated in room temperature (25 ± 2 °C) for seven days^43^. The aliquots were then filtered through filter paper Whatman No.1 and evaporated to dryness using Eyela vacuum rotary evaporator at 40 °C and 173 Hpa. Hot aqueous extracts were prepared by boiling freeze dried samples (100 g/L) at 100 °C for two hours^43^. The aliquots were filtered through filter paper Whatman No.1 and the filtrate was freeze dried.

### Quantification of total phenolic content (TPC)

Total phenolic content was quantified by using the method of Bhanja *et al.*^23^. The extracts ranging from 10–1000 μg/mL were mixed with 50 μL of 10% Folin-Ciocalteu reagent in 96-well microplate and incubated at room temperature for three minutes. Then, 100 μL of 10% sodium carbonate was added and the absorbance was measured at 750 nm after one hour incubation at room temperature. Gallic acid was used as a standard and all data are expressed in mg of gallic acid (GAE)/ g of extracts.

### Antioxidant activity of different extracts of *Ganoderma neo-japonicum*

#### 2.9.1 2,2-diphenyl-1-picrylhydrazyl (DPPH)radical scavenging properties

The various extracts were dissolved in DMSO and diluted to yield concentrations ranging from 1–1000 μg/mL. Diluted extracts (5 μL) was mixed with DPPH reagent (195 μL) in a 96-well microplate and the absorbance was measured at 515 nm every 20 minutes for two hours using a microplate reader. Ascorbic acid was used as a standard and the assays were conducted in quadruplicates. Radical scavenging activities of the various extracts were expressed as percentage (%) of DPPH quenched^44^. IC50 was determined using GraphPad Prism software version 5.0.
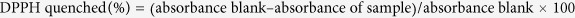


#### 2-azinobis (3-ethylbenzothiazoline-6-sulfonic acid) (ABTS) radical scavenging properties

ABTS radical scavenging assay was carried out according to the method of Bhanja *et al.*^23^. The ABTS reagent was prepared by mixing 5 mL of 7 mM ABTS with 89 μL of 140 mM potassium persulfate. The solution was incubated in the dark, at room temperature for 12 to 16 hours in order to generate ABTS radicals. The radical-containing reagent was then diluted with absolute ethanol to yield an absorbance in the range of 0.68 to 0.72 at 734 nm wavelength. The assay was continued by mixing 10 mL of extract with 100 μL of the appropriately diluted ABTS reagent. The absorbance was measured after one minute at 734 nm using a microplate reader. Trolox was used as a standard and radical scavenging activities of the various extracts were expressed as percentage (%) of ABTS quenched. IC50 was determined using GraphPad Prism software version 5.0.
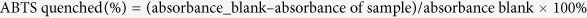


#### Ferric-reducing antioxidant power (FRAP)

Acetate buffer (pH 3.6, 300 m M), 2,4,6-tripyridyl-s-triazine (TPTZ) reagent (10 mM) and ferric chloride (20 mM) were mixed in 10:1:1 ratio (v/v). The various extracts (10 μL) were pipetted into 96-well microplate in quadruplicates, followed by 300 μL of FRAP reagent. The absorbance was measured at 593 nm after four minutes of incubation at room temperature. Ferric sulphate was used as a standard and the data was expressed as μmol Fe^2+^/mg equivalent for each extracts.

### Statistical analysis

Results were from three independent experiments performed in triplicates. All data were subjected to analysis of variance (ANOVA) using Prism Graphpad Statistical Software version 5. The differences between each sample were evaluated by Tukey multiple range test where p < 0.05 was considered significantly different.

## Additional Information

**How to cite this article**: Tan, W.-C. *et al.* *Ganoderma neo-japonicum* Imazeki revisited: Domestication study and antioxidant properties of its basidiocarps and mycelia. *Sci. Rep.* **5**, 12515; doi: 10.1038/srep12515 (2015).

## Figures and Tables

**Table 1:** The medicinal uses of *Ganoderma neo-japonicum* by different tribes in Peninsular Malaysia.

**Part used**	**Symptom/Disease/Application**	**Preparation method**	**Mode of delivery**	**Tribe**
Basidiocarp (Pileus and Stipe)	Overall wellness	Decoction	Oral	Lanoh and Temiar
	Respiratory symptoms	Decocted	Oral	Jahai
	Asthma Lung infection	Decocted	Oral	Kintak
	Diabetes	Decocted	Oral	Temiar and Kintak
	Body and joints’ pain	Decocted	Oral	Temiar
	Cancer	Decocted	Oral	Kensiu and Lanoh
	Fever	Decocted	Oral	Lanoh
	Wound healing	Burnt and mixed with water	Topical (adults only)	Bateq
Stipe	Epilepsy	Cut and strung	Worn round neck	Semai
Pileus	Fever	Hot water infusion	Oral	Temuan and Jakun

**Table 2:** Summary of research on *Ganoderma neo-japonicum* in the past two decades.

**No.**	**Objectives**	**Major finding/s**	**References**
1	To test the ability of *G. neo-japonicum* to produce extracellular enzymes	The mushroom showed strong β-glucosidase and avicelase activities	12
2	To determine the optimal media conditions for the detection of cellulase activity in *G. neo-japonicum*	The dye reagent, pH, and temperature for the optimum detection of cellulase activity for *G. neo-japonicum* were Congo red, pH 7.0, and 25 °C; respectively.	13
3	To investigate the effects of amino acids on the production of total phenolic compounds in the mycelial culture of *G. neo-japonicum*	Tryptophan was the best amino acid in accumulation of total phenolic compounds	14
4.	To investigate the effects of amino acids on the production of ergothioneine (ERG) by using the mycelia culture of *G. neo-japonicum*	Methionine enhanced the ERG production	15
5	To isolate steroids from *G. neo-japonicum*	Two lanostanoids, four ergosteroids and one steroid were isolated	16
6	To study the free radical scavenging and antihepatotoxic activity of *G. neo-japonicum*	The hepatotoxicity of CCl_4_ in rodent liver was significantly reduced	17
7	To study the biosynthesis of cryptoporic acid I and H in *G. neo-japonicum*	Cryptoporic acid I was shown to be biosynthesised *via* cryptoporic acid H and catalysed by cytochrome P-450	18
8	To study the cryptoporic acids present in *G. neo-japonicum*	Cryptoporic acids H and I were isolated and characterised	19
9	To study the effects of *G. neo-japonicum* on diabetic control	The extract enhanced adipogenesis and modulated PPARγ expression in 3T3-L1 cells	9
10	To study the antioxidant activity of *G. neo-japonicum*	The ethanol extract from wheat fermented with this mushroom mycelia showed the most potent antioxidant activities	20
11	To study the neuritogenic effects of *G. neo-japonicum*	The extract enhanced neurite outgrowth activity in PC12 cells	8

**Table 3:** Antioxidant activities of different extracts of *Ganoderma neo-japonicum*.

**Extracts**	**Total phenolic content (Equivalent of μg gallic acid/mg extracts)**	**DPPH radical scavenging properties IC_50_ (μg/ml)**	**ABTS radical scavenging properties IC_50_ (μg/ml)**	**FRAP value (Equivalent of μmol Fe2+/μg extracts)**
Ascorbic acid (standard)	—	13.06 ± 1.28a	—	—
Trolox (standard)	—	—	296.62 ± 1.23a	—
Ethanolic extract of wild basidiocarps*	101.01 ± 0.59a	29.95 ± 1.67b	720.87 ± 3.12b	0.76 ± 0.03a
Hot water extract of wild basidiocarps*	16.08 ± 0.14b	81.83 ± 4.23c	1801.74 ± 6.19d	0.36 ± 0.10b
Ethanolic extract of cultivated basidiocarps¥	10.30 ± 0.12b	2601.38 ± 7.19h	2601.38 ± 7.12e	0.05 ± 0.00b
Hot water extract of cultivated basidiocarps¥	23.76 ± 0.76b	1343.01 ± 7.33g	1343.01 ± 8.21c	0.27 ± 0.00b
Ethanolic extracts of solid substrate fermentation£	10.84 ± 0.34b	274.75 ± 3.22d	3473.3 ± 8.23f	0.07 ± 0.01b
Hot water extract of solid substrate fermentation£	5.35 ± 0.40b	1440.29 ± 7.23g	7005.55 ± 7.59i	0.11 ± 0.07b
Ethanolic extract of myceliaØ	20.55 ± 0.53b	457.45 ± 4.12f	4503.95 ± 8.28g	0.21 ± 0.15b
Hot water extract of myceliaØ	2.32 ± 0.13c	384.68 ± 2.11e	6402.88 ± 7.19h	0.06 ± 0.01b
Filtrate^Ø^	17.80 ± 0.22b	464.8 ± 3.19f	6402.88 ± 7.19h	0.16 ± 0.12b

^*^growing on bamboo basal stem in the forest, ^¥^cultivated on sawdust, ^£^solid substrate fermentation on wheat grains, ^Ø^mycelia and filtrate from submerged culture using complex media as substrate. Values are means of triplicates ± SD from three independent experiments. Different letters (a–i) denote the means were significantly different at p = 0.05.

**Table 4:** Percentage of cellulose, hemicelluloses and lignin content in different agricultural wastes.

**Agricultural wastes**	**Cellulose (%)**	**Hemicellulose (%)**	**Lignin (%)**	**References**
Sawdust	31–64	71–89	14–34	26
Bamboo dust	41–49.1	—	25.2–28.5	27
Oil palm trunk tissue	39.9	21.2	22.6	28
Oil palm leaves	49.8	83.5	20.5	29
Paddy straw	28–48	—	12–16	30
Cotton waste	90	—	<2	31
